# New Model of Action for Mood Stabilizers: Phosphoproteome from Rat Pre-Frontal Cortex Synaptoneurosomal Preparations

**DOI:** 10.1371/journal.pone.0052147

**Published:** 2013-05-14

**Authors:** Maria Corena-McLeod, Consuelo Walss-Bass, Alfredo Oliveros, Andres Gordillo Villegas, Carolina Ceballos, Cristine M. Charlesworth, Benjamin Madden, Paul J. Linser, Leslie Van Ekeris, Kristin Smith, Elliott Richelson

**Affiliations:** 1 Neuropsycopharmacology Laboratory, Mayo Clinic, Jacksonville, Florida, United States of America; 2 Department of Psychiatry, University of Texas Health Science Center, San Antonio, Texas, United States of America; 3 College of Medicine, Health Science Center, Department of Medicine, Division of Hematology/Oncology, University of Florida, Gainesville, Florida, United States of America; 4 Center for Translational Research in Neurodegenerative Diseases, University of Florida, Gainesville, Florida, United States of America; 5 Mayo Proteomics Research Center, Mayo Clinic, Rochester, Minnesota, United States of America; 6 The Whitney Laboratory for Marine Bioscience, University of Florida, St. Augustine, Florida, United States of America; 7 Cancer Biology, Mayo Clinic, Jacksonville, Florida, United States of America; Nathan Kline Institute for Psychiatric Research and New York School of Medicine, United States of America

## Abstract

**Background:**

Mitochondrial short and long-range movements are necessary to generate the energy needed for synaptic signaling and plasticity. Therefore, an effective mechanism to transport and anchor mitochondria to pre- and post-synaptic terminals is as important as functional mitochondria in neuronal firing. Mitochondrial movement range is regulated by phosphorylation of cytoskeletal and motor proteins in addition to changes in mitochondrial membrane potential. Movement direction is regulated by serotonin and dopamine levels. However, data on mitochondrial movement defects and their involvement in defective signaling and neuroplasticity in relationship with mood disorders is scarce. We have previously reported the effects of lithium, valproate and a new antipsychotic, paliperidone on protein expression levels at the synaptic level.

**Hypothesis:**

Mitochondrial function defects have recently been implicated in schizophrenia and bipolar disorder. We postulate that mood stabilizer treatment has a profound effect on mitochondrial function, synaptic plasticity, mitochondrial migration and direction of movement.

**Methods:**

Synaptoneurosomal preparations from rat pre-frontal cortex were obtained after 28 daily intraperitoneal injections of lithium, valproate and paliperidone. Phosphorylated proteins were identified using 2D-DIGE and nano LC-ESI tandem mass spectrometry.

**Results:**

Lithium, valproate and paliperidone had a substantial and common effect on the phosphorylation state of specific actin, tubulin and myosin isoforms as well as other proteins associated with neurofilaments. Furthermore, different subunits from complex III and V of the electron transfer chain were heavily phosphorylated by treatment with these drugs indicating selective phosphorylation.

**Conclusions:**

Mood stabilizers have an effect on mitochondrial function, mitochondrial movement and the direction of this movement. The implications of these findings will contribute to novel insights regarding clinical treatment and the mode of action of these drugs.

## Introduction

Although mitochondrial dysfunction appears to have a strong impact on the pathogenesis of bipolar disorder (BD) and schizophrenia little is known about the effects of impaired mitochondrial movement [1]. Mitochondrial short and long-range movement is necessary to generate the energy needed for synaptic signaling and plasticity [2]. This energy is supplied in the form of Adenosine 5′-triphosphate (ATP) by a series of chemical reactions that take place within the electron transport chain (ETC).

The ETC is organized in an assembly line-like manner within and across the inner mitochondrial membrane (IMM). Three of the complexes (I, III, and IV) pump protons (H^+^) outwardly across the IMM into the inter membrane space to establish the proton gradient necessary for the production of ATP by complex V (ATP synthase or ATPA). Drugs that uncouple the proton gradient across the mitochondrial inner membrane and that inhibit ATPA will therefore regulate mitochondrial movement.

Due to the complexity of neuronal morphologies, an effective mechanism to transport and anchor mitochondria to pre- and post-synaptic terminals is as important as functional mitochondria in neuronal firing and plasticity. Recently our group reported the effects of the mood stabilizer lithium, the anticonvulsant/mood stabilizer valproate, and paliperidone (classified as antipsychotic) on synaptoneurosomal proteins from rat pre-frontal cortex [3]. Pathways affected by both lithium and paliperidone included oxidative phosphorylation, ETC, and post-synaptic cytokinesis, implicating similar effects of these drugs on signaling pathways, energy metabolism, and synaptic plasticity. In this manuscript, we report the effects of chronic lithium, valproate, and paliperidone treatment on the phosphoproteome (group of phosphorylated proteins) at the synaptoneurosomal level and their possible role in mitochondrial movement to the synapse.

## Materials and Methods

Unless otherwise noted, chemicals were obtained from Sigma (St. Louis, MO, USA). Paliperidone was provided by Janssen Ortho McNeil Scientific Affairs. All animal protocols were approved by the Mayo Clinic Institutional Animal Use and Care Committee (IACUC).

### Animal drug treatment

Four male Sprague–Dawley rats (300–400 g) were used per group treatment. The animals were housed in a temperature- and light-controlled room with free access to food and water. In addition, rats receiving lithium chloride were provided with a bottle of 0.9% saline to minimize the electrochemical imbalance caused by the diuretic properties of lithium. All tests were performed during the first half of a 12 h light/dark cycle following approved procedures by the Mayo Foundation Institutional Animal Use and Care Committee. Animals in each treated group were injected intraperitoneally (i.p.) daily for 28 days with 200 µl each of the following: lithium chloride dissolved in 0.9% saline (22 mg/kg), valproic acid in saline (200 mg/kg), and paliperidone (1 mg/kg) in 0.3% D,L-tartaric acid dissolved in saline (pH 4.0 adjusted with 1 M NaOH) as previously described [6]. This administration paradigm has previously been used in rats to achieve blood levels within the therapeutic range seen in humans (0.5–1.2 mM) [92]–[94]. Two groups were simultaneously injected with equal volumes of vehicle as controls: 0.9% saline and 0.3% tartaric acid in saline (pH adjusted to 4.0 with 1 M NaOH). The exact dose for each drug was achieved regardless of weight gain of the animals by weighing the rats every week and adjusting the amount of drug in solution accordingly. Animals were decapitated 24 h following last drug administration (between 0900 and 1200) and their brains were dissected on ice. PFC was collected and snap-frozen on dry ice as described by Tilleman [4].

### Synaptoneurosomal preparations

Synaptoneurosomal-enriched fractions were prepared as previously described [3]. Briefly, PFC from each treated rat and control was weighed, homogenized in 500 µl synaptoneurosome buffer (10 mM HEPES, 1 mM EDTA, 2 mM EGTA, 0.5 mM DTT, 10 µg/ml Leupeptin and 50 µg/ml soybean trypsin inhibitor, pH 7.0) at 4°C with the use of a Teflon-glass mechanical tissue grinder (0.25 mm clearance). The homogenate was diluted further with the same volume of synaptoneurosome buffer and briefly and gently sonicated with the use of a 60 sonic dismembrator (Fisher Scientific, Pittsburg, PA) delivering 3 pulses with an output power of 1 (dial setting 1). The resulting solution was loaded into a 3 ml syringe and filtered once through one layer of pre-wetted 180 µm pore nylon filters (Millipore, Billerica, MA), held in a 13 mm diameter filter holder. The filtrate was loaded into a 5 ml syringe and filtered once through a 60 µm pore nylon filter (Millipore), divided into small fractions and filtered through a 5 µm filter (National Scientific Company, Rockwood, TN), pooled and centrifuged at 100,000×g for 10 min. An aliquot of the supernatant was saved for Western blot analysis. The resulting pellet containing the synaptoneurosomal-enriched preparation was re-dissolved in a small amount of synaptoneurosome buffer and centrifuged again at 100,000×g for 10 min. These synaptoneurosomal-enriched pellets were saved for further analysis. Aliquots of the initial protein homogenate and the filtrate from each step were saved to determine protein concentration and for Western blot analysis to confirm the presence of synaptoneurosomal proteins. Protein concentration was determined with the use of the BCA assay (Pierce, Rockford, IL) and samples were stored until analyzed by two-dimensional fluorescence differential gel electrophoresis (2D-DIGE).

### Western blots

Western blots (two dimensions) were used to confirm phosphorylation and identification of synaptoneurosomal and mitochondrial proteins after treatment. Proteins extracted above were separated with the use of 2D-DIGE and then transferred to a Nylon membrane (Millipore, Billerica, MA), at 30 V and 4°C for 4 h. After transfer was completed, the blot was rinsed with Tris-buffered saline (TBS) pH 7.4, incubated for 30 min in blocking solution (20 g dry powdered milk, 400 ml 1× TBS, 400 µl goat serum, and 200 µl Tween 20) followed by three consecutive washes of 15 min each with TBS. After rinsing, the blot was incubated in primary antibodies in blocking solution as described by Villasana and collaborators [5]. Phospho-antibodies included anti-phospho-tubulin and anti-phospho actin at 1∶1,000 dilution (Applied Biomics, Hayward, CA). Horseradish peroxidase-conjugated secondary antibodies (goat anti-mouse) diluted at 1∶2,000 were used to bind the primary antibodies. Blots were washed in TBS three consecutive times for 5 min each and visualized with the use of Enhanced Chemi-Luminescence (ECL) Western blotting detection from Amersham Biosciences (Piscataway, NJ, USA) and Kodak Biomax film (Kodak, Rochester, NY) or following Applied Biomics protocols.

### 2D-DIGE and phosphorylation

2D-DIGE was performed by Applied Biomics (Hayward, CA) following established protocols. In detail, 2D lysis buffer (30 mM Tris–HCl, pH 8.8, 7 M urea, 2 M thio-urea, and 4% CHAPS) was added to each synaptoneurosomal pellet and sonicated for 5 s with the use of VirSonic 100 (VirTis) at power level 4. After vigorous shaking at RT for 30 min, the lysates were cleared at 16,000×g for 30 min. Supernatant was transferred to Eppendorf tubes. Protein concentration for each sample was adjusted to 5 mg/ml for with lysis buffer. 30 µg of synaptoneurosomal preparation was labeled with 0.7 µl CyDye dilution (Cy2, Cy3, and Cy5, Amersham, Piscataway, NJ) per group. The CyDyes were diluted 1∶5 with dimethylformamide (DMF) before each reaction, incubated on ice for 30 min, followed by addition of 0.7 µl of 10 mM lysine to stop the labeling reaction. The final mix was kept on ice in the dark for 15 min. The CyDye-labeled preparations were mixed and an equal volume of 2×2D sample buffer (8 M urea, 4% CHAPS, 20 mg/ml DTT, 2% pharmalytes, and trace amount of bromophenol blue) was added. This was followed by the addition of 100 µl destreak solution (GE Healthcare, Piscataway, NJ) and rehydration buffer (7 M urea, 2 M thiourea, 4% CHAPS, 20 mg/ml DTT, 1% pharmalytes and trace amount of bromophenol blue) to a total volume of 260 µl. Solutions were incubated at RT for 10 min on a shaker and centrifuged for 10 min at 16,000×g before loading 250 µl per immobilized pH gradient (IPG) strip [13 cm, pH 3–10 linear isoelectric focusing (IEF) strip, from Amersham]. IEF was performed for a total of 25000 V-h under standard conditions (Amersham). Each IPG strip was incubated with 10 ml of equilibration solution 1 (50 mM Tris–HCl, pH 8.8, containing 6 M urea, 30% glycerol, 2% SDS, trace amount of bromophenol blue, and 10 mg/ml DTT) for 15 min with gentle shaking followed by incubation in 10 ml of equilibration solution 2 (50 mM Tris–HCl, pH 8.8, containing 6 M urea, 30% glycerol, 2% SDS, trace amount of bromophenol blue, and 45 mg/ml iodoacetamide) for 10 min with gentle shaking. Strips were rinsed in SDS gel running buffer once and inserted into a 10.5% SDS gel prepared on low fluorescent glass plates (18×16 cm, 1 mm thickness) and sealed with 0.5% agarose sealing solution in SDS running buffer. Electrophoresis was performed at 16°C. Each gel was scanned immediately following electrophoresis with a Typhoon Trio Scanner (Amersham). Images were analyzed with ImageQuant software. Phosphorylation was detected with Pro-Q® Diamond stain (Invitrogen). Pro-Q® Diamond phosphoprotein gel stain was used in conjunction with SYPRO® Ruby protein gel stain for total-protein stain. Gels were stained according to the manufacturer's recommendation. Gels were fixed in fix solution (50% methanol, 10% acetic acid) for 1 h and then again in a fresh fix solution overnight at RT. The next day, gels were washed three times with water for 15 minutes, stained with ProQ Diamond Phosphoprotein Gel Stain for 2 h with gentle agitation in the dark and de-stained three times in de-stain solution (20% acetonitrile, 50 mM sodium acetate, pH 4.0) for 30 min. The stained gels were washed twice with water for 5 minutes and scanned with Typhoon Trio with the use of excitation 532 nm laser. The ratio of Pro-Q® Diamond dye to SYPRO® Ruby dye signal intensities for each band or spot was calculated. This provided a measure of the phosphorylation level normalized to the total amount of protein. Using both stains in combination, it was possible to distinguish a lightly phosphorylated, high-abundance protein from a heavily phosphorylated, low-abundance protein.

### Image analysis and spot detection

Further analysis and quantification were done with DeCyder software (Ludesi, Lund, Sweden). Spot detection and matching were performed by the software and each spot was manually checked for accuracy. Differences in protein expression were determined with Ludesi's 2D Interpreter. Minimum protein volume was set at 200 and only those proteins with a 2 fold or more difference in protein expression, a 100% presence in all gel images, and P-values<0.05 (ANOVA) were selected. Hierarchical clustering (HC) and Support Vector Machines (SVM) were used to classify selected proteins into groups. Groups of images were created per treatment group. Analysis of expression was performed independently by comparing each treatment separately and all sets of gels to the saline control group. The paliperidone, risperidone, and saline groups were compared to the tartaric acid group to determine changes in expression caused by the addition of acid to aid in solubilization of the drugs. Unsupervised and supervised HC were performed. For the supervised HC, 75% of the 20 gel images in each group were used as training sets and all gels were analyzed as test sets. Results of both supervised and unsupervised HC were compared and a list of proteins of interest (POI) was generated. Pearson correlation and average linking methods were used to determine distance function and to cluster the classifying proteins. One degree polynomial analysis was used for SVM. Protein spots showing significant changes in mean normalized quantity from the POI were selected. Each spot was verified by manual comparison of the three sets of gels before being picked and identified by nano LC-MS/MS. Protein spots were excised from the gel with the use of an Ettan spot picker (Amersham) and sent to the Proteomics Research Center at the Mayo Clinic in Rochester, MN for further analysis or identified by Applied Biomics as described next.

### Trypsin digestion and nanoLC-ESI tandem mass spectrometry

Excised spots were reduced with 20 mM DTT/50 mM Tris–HCl, pH 8.1 at 55°C for 30 min and alkylated with 40 mM iodoacetamide at RT for 30 min in the dark. Proteins were digested *in-situ* with 10 µl (0.002 µg/µl) trypsin (Promega Corporation, Madison, WI) in 20 mM Tris–HCl, pH 8.1/0.0002% Zwittergent 3–16, at 37°C overnight followed by peptide extraction with 20 µl of 2% trifluoroacetic acid and 40 µl of acetonitrile. Pooled extracts were concentrated to less than 5 µl on a SpeedVac spinning concentrator (Savant Instruments, Holbrook, NY) and then brought up in 0.1% formic acid for protein identification by nano-flow liquid chromatography, electrospray tandem mass spectrometry (nano LC-ESIMS/MS) with the use of a ThermoFinnigan LTQ Orbitrap Hybrid Mass Spectrometer (ThermoElectron, Bremen, Germany) coupled to an Eksigent nanoLC-2D HPLC system (Eksigent, Dublin, CA). The peptide mixture was loaded onto a 250 nl OPTI-PAK trap (Optimize Technologies, Oregon City, OR) custom packed with Michrom Magic C8 solid phase (Michrom Bioresources, Auburn, CA) and eluted with a 0.2% formic acid/acetonitrile gradient through a Michrom packed tip capillary Magic C18 column (75 µm×150 mm). The LTQ Orbitrap mass spectrometer performed a Fourier Transformed (FT) full scan from 380–1600 m/z with resolving power set at 60,000 (400 m/z), followed by linear ion trap MS/MS scans on the top 3 ions. Dynamic exclusion was set to 2 and selected ions were placed on an exclusion list for 60 s. The MS/MS raw data were converted to DTA files with the use of ThermoElectron Bioworks 3.2 and correlated to theoretical fragmentation patterns of tryptic peptide sequences from the Swissprot databases with the use of both SEQUEST™ (ThermoElectron, San Jose, CA) and MASCOT™ (Matrix Sciences London, UK) search algorithms running on 10 node clusters. All searches were conducted with fixed cysteine modifications of +57 for carboxamidomethyl-cysteines and variable modifications allowing +16 with methionines for methionine sulphoxide, and +42 for protein N-terminal acetylation. The search was restricted to trypsin generated peptides allowing for 2 missed cleavages and was open to all species. Peptide mass search tolerances were set to 10 ppm and fragment mass tolerance was set to ±0.8 Da. A protein was considered identified when both Mascot and Sequest gave at least two consensus peptides with individual cross correlation scores exceeding 2.2 for +2 peptides or 3.2 for +3 peptides and 95% probability scores in addition to ranking as the number one hit for their respective MS/MS spectra.

### Criteria for protein identification

Scaffold (version Scaffold-2_00_02, Proteome Software Inc., Portland, OR) was used to validate MS/MS based peptide and protein identifications. Peptide identifications were accepted if they could be established at greater than 95% probability as specified by the Peptide Prophet algorithm [6]. Protein identifications were accepted if they could be established at greater than 95% probability and contained at least 2 identified peptides. Protein probabilities were assigned by the Protein Prophet algorithm [7]. Proteins that contained similar peptides and could not be differentiated based on MS/MS analysis alone were grouped to satisfy the principles of parsimony.

### Mitochondria and SYN1 staining in cell culture

Rat primary neuronal cells from PFC obtained from BrainBits (Springfield, IL) were cultured at 0.1 million/ml/well in Neurobasal/B27/0.5 mM Glutamax culture medium in poly-D-lysine (0.15 ml/cm^2^, 50 µg/ml water) glass coated disks inside 24 well plates and incubated at 37°C in 5% CO_2_, 95% O_2_ atmosphere following vendor instructions. Medium was changed every three days. Differentiated primary neurons (after 4 d) were treated with lithium chloride (0.1, 1.0 and 10 mM), valproate (0.05, 0.5, and 5 mM), or paliperidone (0.1, 1.0, and 10 mM) in 0.3% D,L-tartaric acid dissolved in saline (pH 4.0 adjusted with 1 M NaOH). Treated cells were incubated in different concentrations of MitoTracker CMXRos ® red dissolved in dimethylsulfoxide (DMSO) until the optimum conditions for incubation without interfering with cell viability were determined. Calcein was used to determine cell viability after treatment (not shown). A final concentration of 50 nM for 15 min was optimum to visualize active mitochondria (Invitrogen, Carlsbad, CA). We have previously reported that expression of SYN1 in rat synaptoneurosomal preparations from PFC was increased after valproate, lithium, and paliperidone treatment [3]. Therefore, synapses were visualized with SYN1 antibodies. To detect SYN1, cells were rinsed twice with PBS, fixed with 4% paraformaldehyde and incubated in MitoTracker, CMXRos ®. Nuclei were stained with the use of Hoechst or DAPI nuclear stain at 1∶1,000 dilution. Primary antibodies were used at a concentration of 1∶500. Secondary antibody (Alexa 488) was used at a concentration of 1∶1,000. Controls included saline and 0.3% D,L-tartaric acid dissolved in saline (pH 4.0 adjusted with 1 M NaOH).

### Mitochondrial staining in human SKNSH cells

Human neuroblastoma cells (SK-N-SH) obtained from ATCC (Manassas, VA) were maintained in Minimum Essential Medium (MEM) supplemented with 10% heat-inactivated Fetal Bovine Serum (FBS), penicillin (45 µg/ml), streptomycin (45 µg/ml), MEM Non-Essential Amino Acids (90 µM), and pyruvic acid (90 µM) in plastic Corning flasks at 37°C under 5% CO_2_/95% air. All procedures were carried out at the University of Texas Health Science Center (San Antonio, TX). Medium was changed 3 times per week. Confluent cultures were washed with phosphate-buffered saline (PBS), followed by treatment with lithium (1, 10, or 20 µM), paliperidone (1, 10, or 50 µM), clozapine (10, 20 or 50 µM), haloperidol (10, 30 or 50 µM) or vehicle (saline) for 24 h. Experiments were performed in triplicate with all drug concentrations. Cells were seeded onto 8-well chambered cover glasses. To visualize mitochondrial morphology, cells were incubated with 100 nM MitoTracker® Green (Invitrogen, Eugene, OR, USA) for 1 h at 37°C under 5% CO_2_/95% air. Confocal images were obtained with the use of an FV 1000 imaging system (Olympus America, Center Valley, PA) mounted on an Olympus IX-81 research grade microscope. The lens used for imaging was a PlanApo 60X (Olympus America, Center Valley, PA, USA), 1.4 oil immersion. Acquisition conditions were determined for each imaging channel with the use of individually labeled fluorescent samples.

### MitoTracker® stain of live tissue

To determine the effect of the drugs in mitochondria in live tissues, animals were sedated 1 h after the last injection with the use of CO_2_ and quickly decapitated. The PFC was rapidly sliced in PBS with the use of a McIlwain Mechanical Tissue Chopper. Slices (50 µm thick) were incubated for 10 min in 50 nM MitoTracker® red dissolved in DMSO and PBS (stock solution made in DMSO and dilution made in PBS). After MitoTracker® staining, tissue was rinsed twice with PBS and fixed in 4% paraformaldehyde in PBS. Images were obtained with the use of confocal scanning microscopy. Nuclei were stained with DAPI.

## Results

Our results support our hypothesis that mood stabilizer drugs promote migration of mitochondria to the synapse in order to provide energy for neurotransmission. Evidence of mitochondrial movement included proteomics results and visualization of mitochondrial movement in cell culture and rat PFC tissue.

### 2D-DIGE protein phosphorylation

Staining with ProQ Diamond indicated that specific cytoskeletal, mitochondrial, and regulatory proteins were highly phosphorylated (≥7 fold) in response to paliperidone, lithium, and valproate chronic treatment. Table 1 lists these proteins classified according to treatment. Lithium and valproate treatment resulted in phosphorylation of similar proteins as shown in Table 1. Different isoforms of actin, including actin cytoplasmic 1 (ACTB), actin (alpha skeletal muscle by homology or ACTS), and tubulin isoform tubulin alpha-1b-chain (TBA1B) were highly phosphorylated after treatment with these drugs. In addition, proteins involved in synaptic plasticity and mitochondrial transport were also heavily phosphorylated after mood stabilizer treatment. Isoforms of myosin heavy chain (MYH1, MYH2, and MYH6) involved in actin-based motility were phosphorylated in all groups compared to the saline and tartaric acid controls. Alpha internexin (AINX), a key structural protein, was heavily phosphorylated in the paliperidone treated group along with another structural protein homologous to desmoplakin (DESP). Interestingly, the vacuolar ATP synthase catalytic subunit A (ATP6V1A) was phosphorylated after valproate and lithium treatment, while the vacuolar ATP synthase catalytic subunit B (ATP6V1B2) was phosphorylated after paliperidone treatment. Other mitochondrial proteins that showed increased phosphorylation included alpha-enolase (ENOA) and fructose-bisphosphate aldolase A (ALDOA). Ubiquinol-cytochrome c reductase complex core protein 1 (UQCRC1 from complex III) was equally phosphorylated by all three drugs although a different sequence was found to be phosphorylated only by paliperidone. This sequence was identified as Cytochrome b-c1 complex subunit 1 (QCR1), which is the same as UQCRC1. Other proteins highly phosphorylated in the paliperidone chronic treatment group included heat shock cognate 71 kDa (HSP7C), HSP90B, 78 KDa Glucose regulated protein precursor (GRP78), a protein homologous to protein kinase C inhibitor (1433Z), and homer protein homolog 1 (HOME1).

**Table 1:** Protein phosphorylation in synaptoneurosomal preps from rat PFC as a result of chronic lithium, valproate, and paliperidone treatment.

VALPROATE AND Li TREATMENT						
FUNCTION	Spot ID	Protein	MW (kDa)	N	% SC	Peptide sequence
STRUCTURE	9707	ACTB	41,784.90	10	35.2	AGFAGDDAPR
ACTIN BASED MOTILITY	9529	MYH1	223,132.50	21	12.2	DFEISQIQSK
	9901	MYH2	223,132.50	56	31.4	ADIAESQVNK
	10433	MYH1	223,132.50	37	20.8	DFEISQIQSK
	9680	MYH6	223,132.50	28	15.7	DLEEATLQHEATAAALR
	10280	MYH6	223,132.50	16	10.4	AITDAAMMAEELKK
PALIPERIDONE TREATMENT						
MITOCHONDRIA	9855	ATP6V1A	68,309.40	19	41.3	ADYAQLLEDMQNAFR
STRUCTURE	9286	ACTB	41,784.90	30	70.9	AGFAGDDAPR
	10319	ACTB	41,784.90	27	72.3	AGFAGDDAPR
	9881	ACTS	42,044.20	15	36.1	CDIDIR
	10445	TBA1B	50,133.70	13	32.4	AVCMLSNTTAIAEAWAR
	9452	AINX	56,098.70	30	65.9	ALEAELAALR
	9347	AINX	56,098.70	30	64.8	ALEAELAALR
	9899	DESP	331,763.40	20	11.3	AEMDMVAWGVDLASVEQHINSHR
MOTILITY	9943	MYH1	223,132.50	30	16.8	DALISQLSR
	9322	MYH1	223,132.50	22	11.9	DALISQLSR
MITOCHONDRIA	10362	ALDOA	39,334.50	27	64.3	AAQEEYIK
	9307	ATP6V1B2	56,534.30	21	58.7	AVVGEEALTSDDLLYLEFLQK
	10379	ENOA	47,111	23	62	AAVPSGASTGIYEALELR
	9294	UQCRC1	52,830.60	14	39.6	EMQENDASMQNVVFDYLHATAFQGTPLAQAVEGPSENV
OTHERS	9425	HSP7C	70,854.70	19	38.5	DAGTIAGLNVLR
	9303	HSP7C	70,854.70	28	48.6	ARFEELNADLFR
	9287	HSP7C	70,854.70	31	59.1	ARFEELNADLFR
	10055	HS90B	83,266.30	24	39.8	ADHGEPIGR
	10040	GRP78	72,330.50	22	45.9	AKFEELNMDLFR
REGULATION	10410	1433Z	27,753.90	26	78	DICNDVLSLLEK
	9831	HOME1	41,287.40	17	55.5	AEPAQNALPFSHSAGDR
ALL TREATMENTS						
MITOCHONDRIA	9628	UQCRC1	52,830.60	26	64.8	ALSKDLPK

All proteins shown in this table exhibited a protein identification probability of 100% and were chosen based on the following criteria: minimum protein volume was set at 200 and only proteins with a 2 fold or more difference in protein expression, a 100% presence in all gel images, and P-values<0.05 (ANOVA) were selected. (%SC = percent sequence coverage, N = number of unique peptides).

### Actin and tubulin

**Figure 1:**
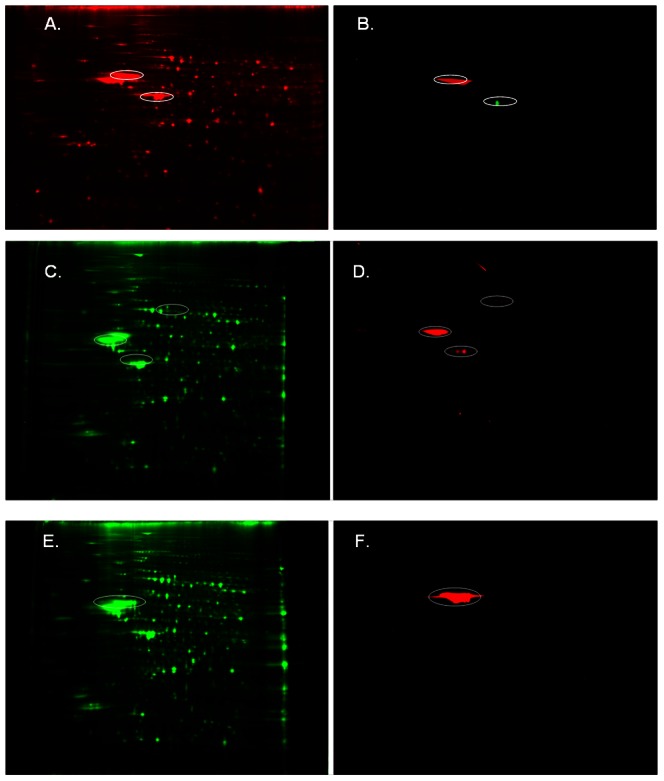
Actin and tubulin were phosphorylated after mood stabilizer treatment. A. 2D-DIGE gel image stained with Sypro Ruby stain showing phosphorylation of synaptoneurosomal preparations of saline treated animals (controls). B. location of actin identified spots (green spot) and tubulin (red spot) in saline treated preparations. C. Spots identified with actin antibodies during Western blots (circled) of synaptoneurosomal preparations after mood stabilizer treatment. D. Western blot identification of phosphorylated actin (circled). E. 2D-DIGE gel image showing phosphorylation after mood stabilizer treatment and the location of tubulin identified spots as indicated by circle. F. Spots identified with tubulin antibodies (circled).

Mitochondrial movement is regulated by actin and tubulin phosphorylation. Protein identification by nano-LC-MS indicated that different isoforms of actin and tubulin were phosphorylated (ACTB, ACTS, and TBA1B). Western blots with the use of anti-phospho actin and anti-phospho tubulin antibodies validated these results and indicated different degrees of phosphorylation according to observed pI values on the gels. The extent of phosphorylation appeared more pronounced in tubulin than in actin isoforms (Figure 1).

### Cell culture

**Figure 2:**
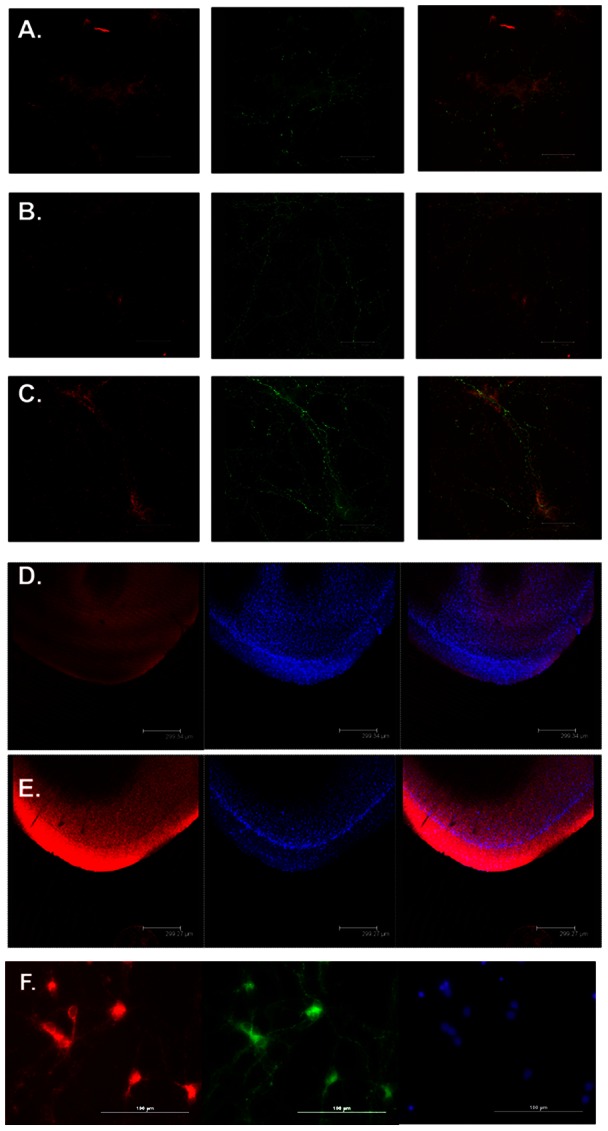
Mood stabilizer treatment resulted in increased mitochondrial transport to the synapse in cell culture as indicated by increased staining of neuronal processes and rat PFC tissue. From left to right: MitoTracker (red), SYN1 (green), and superimposed images (red and green). A. Saline control, B. Lithium (1 mM) treated cells, C. Paliperidone (10 µM) treated cells, D. Saline treated rat PFC tissue slice (left to right: MitoTracker red, DAPI nuclear stain and superimposed image), E. Lithium (22 mg/Kg) treated rat PFC tissue slice (left to right: MitoTracker red, DAPI and superimposed image). F. A close-up of localization of Mito Tracker red (red, left) and SYN1 (green) in paliperidone (0.1 µM) treated cells. Nuclei shown in blue (Secondary antibody: Alexafluor488 Mouse 1∶1000, Mitotracker CFX-Ros 50 nM, Hoecsht nuclear stain 1∶1000).

Our phosphoproteomics studies from rat PFC suggested high levels of phosphorylation of actin and tubulin isoforms as well as other proteins involved in mitochondrial movement. In an attempt to observe changes in mitochondrial movement after treatment with paliperidone, lithium, and valproate, living cells were treated with these drugs at different concentrations. MitoTracker CMXRos ® was used to stain live mitochondria. Our cell culture results indicated an increase in mitochondrial staining after paliperidone treatment compared to that for the saline and tartaric acid controls, suggestive of increased numbers of mitochondria or increased uptake of MitoTracker (Figures 2A, 2B, and 2C). Although the effect of lithium was also marked, the most intense staining was observed with paliperidone. Valproate appeared to have a detrimental effect on the cells at the concentrations tested as indicated by changes in cell and mitochondrial morphology, as well as cell survival (results not shown).

### Staining of mitochondria in PFC tissue

The most noticeable change observed in terms of mitochondrial staining in rat PFC tissue compared to that for the saline control was observed in the animals treated with lithium (Figures 2D and 2E).

### Staining of mitochondria in SKNSH cell

**Figure 3:**
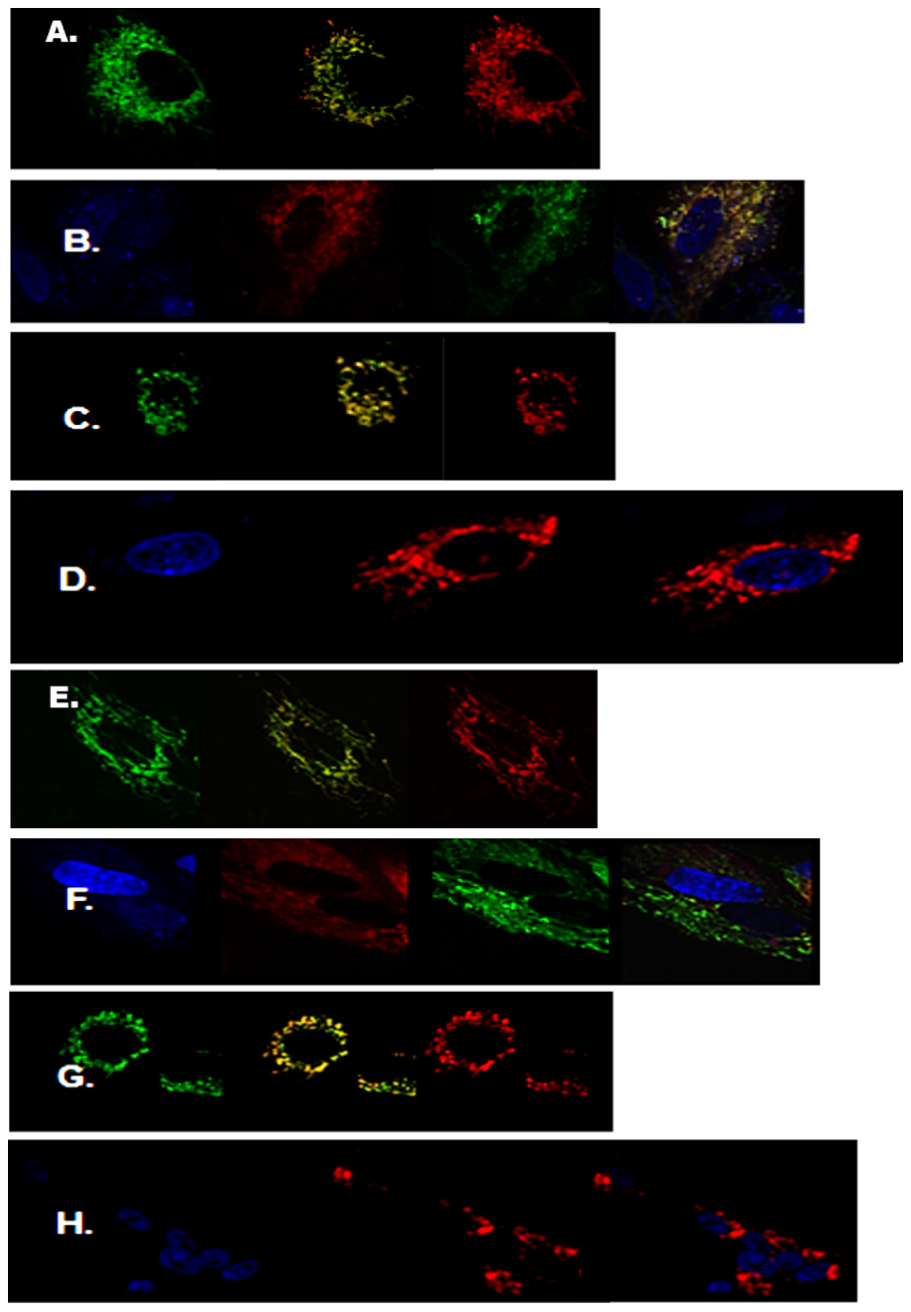
Mitochondrial staining with MitoTracker® green (total mitochondria) and MitoTracker red® (active function mitochondria) shows different morphology in cells at a given time indicative of rapid changes in movement and mitochondrial function as exemplified by the saline controls (A, B, C and D). Anterograde mitochondrial movement (away from the cell nuclei) was observed after treatment with 20 µM lithium (E) and 50 µM Paliperidone (F) as indicated by long filamentous mitochondria. Ballooning and concentration around cell nuclei (blue) was observed after treatment with 50 µM Clozapine (G). Detrimental effects were observed in mitochondria after treatment with 50 µM Haloperidol (H). Superimposed images shown in yellow.

As observed in Figure 3, there are significant differences between the cells treated with saline (3A, 3B, 3C, 3D) and each one of the drugs used. Lithium (3E) and paliperidone (3F) at all concentrations appeared to increase mitochondrial activity and movement away from the cell nuclei as indicated by the presence of filamentous mitochondria. The effect was more pronounced at the highest concentrations shown. Clozapine on the other hand resulted in a ballooning effect of mitochondria with congregation around the nuclei (3G). Treatment with haloperidol at all concentrations resulted in detrimental effects on mitochondria as shown in figure 3H.

## Discussion

Synaptoneurosomes are specialized asymmetric cell-adhesion junctions [8]. Their functionality depends heavily on plasticity, which in turn is determined by expression and organization of cytoskeletal proteins. Although a relationship between cytoskeletal proteins and the role of phosphorylation at the synapse has not been studied in detail, there is a large amount of evidence implicating cytoskeletal protein dysfunction (in particular microtubules) in schizophrenia and affective disorders [9]–[13].

Phosphorylation of ACTB, ACTS, and myosin heavy chains as found in our studies has been previously related to anterograde movement of mitochondria [14] and it has also been demonstrated that actin phosphorylation and tubulin are essential for axonal mitochondrial transport [15]. It has been reported that pre-synaptic axons of lamprey contain two distinct types of mitochondria: small “synaptic” mitochondria, located near neurotransmitter release sites, and larger mitochondria located in more central parts of the axon [16].These results combined with our proteomics results led us to hypothesize that mood stabilizers promote mitochondrial migration to pre and post-synaptic terminals where they influence mitochondrial function, neurotransmitter release and uptake.

During protein phosphorylation, a phosphate group is added to specific amino acids within a protein, changing its conformation, activity and interaction with other proteins. Phosphorylation is the most efficient regulatory mechanism for organelle movement and function within the neuron. Our results indicate that chronic treatment with lithium, valproate, and paliperidone resulted in high levels of phosphorylation of cytoskeletal and mitochondrial proteins in synaptoneurosomal preparations of the rat PFC. We have previously reported that paliperidone and lithium shared similar protein expression profiles in synaptoneurosomal preparations from rat PFC [3].

Every active synapse includes a cluster of synaptic vesicles, a pre-synaptic active zone and a post-synaptic dense matrix [17]. Connected to most synaptic vesicles at the pre-synapse are fine, short filaments of a phosphoprotein called synapsin 1 (SYN1), which modulates neurotransmitter release and facilitates the formation of a lattice of interlinked synaptic vesicles [18]–[23]). Our previous results showed that SYN1 expression is significantly up-regulated by lithium, valproate, and paliperidone in synaptoneurosomal preparations from rat PFC [3]. Although high levels of SYN1 phosphorylation were not detected, this protein is often associated with actin filaments (F-actin) throughout pre-synaptic terminals [24].

Actin is enriched and often co-localizes within the region surrounding the synaptic vesicle cluster [25], [26], [27]. It also constitutes an important component of the active zone [28]. Unlike F-actin, its globular form is distributed homogeneously throughout axonal and dendritic processes in hippocampal neurons suggesting its main role at the synapse is to maintain the synaptic F-actin pool [29]. Our findings indicate that two actin isoforms were differentially phosphorylated after mood stabilizer treatment. Treatment with paliperidone resulted in phosphorylation of ACTB and ACTS while treatment with lithium or valproate resulted in phosphorylation of ACTB only. It has been demonstrated that actin remodeling plays a significant role in the formation of dendritic spines [30], [31]. These dynamic changes are promoted by polymerization from the G-form (monomer) to the F-form (polymer), which occurs only through phosphorylation. Actin fiber formation promotes the changes that ultimately result in long term potentiation (LTP) [31]. Lack of actin polymerization results in reduction in the efficacy of synaptic neurotransmission or long term depression (LTD) that lasts hours or even longer [95]. Heavy actin phosphorylation by mood stabilizers would lead to increased fiber formation, long term enhancement of neurotransmission, and association with SYN1 to interlink and dock synaptic vesicles contributing to the modulation of neurotransmitter release.

**Figure 4:**
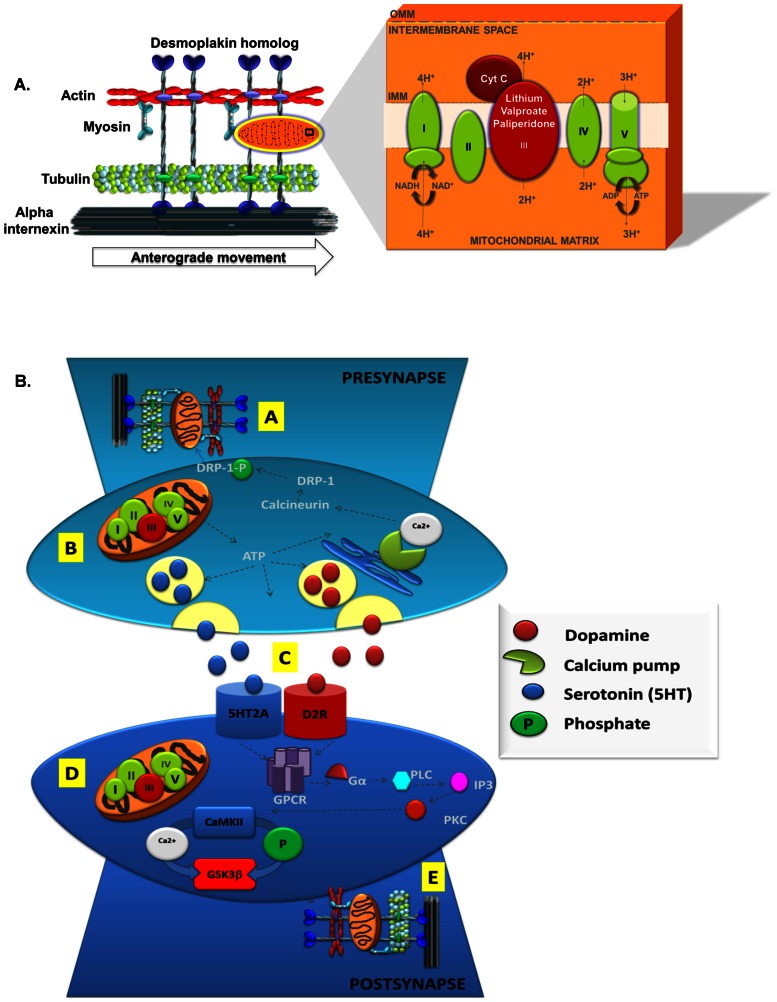
Simplified scheme to illustrate the effects of lithium, valproate and paliperidone treatment on mitochondrial and cytoskeletal protein phosphorylation with effects on mitochondrial transport towards the synapse. A. Cytoskeletal proteins (desmoplakin homolog, myosin, actin, tubulin and alpha internexin) are phosphorylated and involved in the mechanism of anterograde mitochondrial transport. Actin fiber formation is promoted by phosphorylation of actin isoforms. Actin binding domains indicated as purple ovals contribute to network formation through binding of desmoplakin homolog to actin. Phosphorylation of tubulin isoforms promotes microtubule formation and binding to desmoplakin homolog is facilitated through tubulin binding domains (green ovals). Tubulin in turn binds alpha internexin. Anterograde mitochondrial movement is possible through Myosin interaction with actin. Inside the mitochondria (insert), UQCRC1 from complex III (red) was phosphorylated after treatment with all three drugs with effects on mitochondrial function. B. Schematic representation of the effects of anterograde mitochondrial movement on synaptic plasticity and neurotransmission. Each step where the drugs have effects is indicated by a yellow square and a letter inside. These events may or may not happen simultaneously. A. Anterograde mitochondrial movement to the pre-synapse is promoted by all three drugs. B. Mitochondrial function near SNARE complexes provides necessary ATP for neurotransmitter release as well as regulation of Ca^2+^ levels. Myosin heavy chain also plays a role on synaptic vesicle opening. Ca^2+^ affects calcineurin which in turns affects DRP-1. Phosphorylation of DRP-1 influences mitochondria. C. Serotonin and dopamine release is regulated by 5HT2A and D2 receptors. The ratio of serotonin (5HT) and dopamine (DA) available regulates mitochondrial movement to the post-synapse as serotonin promotes anterograde movement while dopamine inhibits it. D. Mitochondrial function at post-synaptic terminals contributes to Ca^2+^ homeostasis influencing CAMKII and GSK3β E. Mitochondrial transport to the post-synapse results in increased levels of ATP necessary for proper functioning of post-synaptic machinery. The number of mitochondria is directly and reciprocally related to the number of active synapses (Li et al., 2004).

In addition to SYN1 and F-actin, microtubules and fodrin (a brain isoform of spectrin) are present at pre-synaptic terminals. Microtubules in particular have been localized at the center of pre-synaptic terminals [32] as exemplified in our model shown in Figure 4A. Microtubules are polymers whose basic units are pairs (dimers) of similar but not identical alpha and beta tubulin. During polymerization, these dimers stack end to end to make a protofilament. We previously found that expression of beta tubulin isoforms was affected by treatment with lithium and paliperidone [6]. In particular, tubulin beta chain (TBB) expression decreased two fold after treatment with lithium while tubulin beta 2C chain (TBB2C) expression increased by two fold in response to paliperidone treatment [6]. Our current studies indicate that TBA1B (or TUBA1B) was highly phosphorylated after treatment with paliperidone. The relevance of phosphorylation of this particular subunit at the synapse is unknown. However, differences in expression and post-translational modification of more than one isoform of alpha and beta tubulin subunits has led to the suggestion that subunit heterogeneity may be in part responsible for the regulation of microtubule assembly and function *in vivo* since as many as 17 alpha and beta tubulin isoforms have been observed in the vertebrate brain [33] and as many as six tubulin isoforms have been observed in single neuronal cells in culture [34]. Our proteomics studies support involvement of different tubulin isoforms in the mechanism of action of mood stabilizers and highlight phosphorylation as an important post-translational modification associated with this process at synaptic terminals.

The evidence linking tubulin and actin to bipolar disorder (BD) or other affective disorders in human brain is plentiful; however, studies linking phosphorylation of these proteins and synaptic function are scarce. Differences in actin expression have been only recently related to BD. As an example, cardiac alpha type has emerged as a novel target linked to susceptibility of BD [35]. Evidence of actin and tubulin phosphorylation in motor axons in vertebrates has not been demonstrated and their role in an invertebrate animal model has only been reported recently. In 2009, it was demonstrated that actin and tubulin phosphorylation decreased during low-frequency depression (LFD) in motor axons and nerve terminals in crayfish. Tubulin and phospho-actin immunoreactivity in pre-synaptic terminals was reduced after LFD. These investigators concluded that de-phosphorylation of pre-synaptic actin and tubulin and associated changes in the cytoskeleton may regulate LFD [36]. Additionally, TBB2C and TBA1B have been found in docked synaptic vesicle fractions from rat brain indicating their importance at the pre-synapse [37].

Although phosphorylation or changes in fodrin expression after treatment with Li, valproate, and paliperidone were not found, it is clear to the authors that actin and tubulin phosphorylation plays a key role in the mechanism of action of mood stabilizers.

Once mitochondria are transported to the pre-synaptic terminal, the produced ATP is used to fuel many processes related to neurotransmitter loading of vesicles, docking, and neurotransmitter release (Figure 4). Mitochondria must be positioned near the synaptic vesicle lattice to facilitate loading of neurotransmitters inside the vesicles. Acidification of the synaptic vesicle lumen by the large multi-subunit vacuolar proton pump (V-ATPase) is required for loading with neurotransmitters [38]. This process is critically dependent on the presence of a functional proton transporting V-ATPase, which not only acidifies the lumen but also creates a voltage gradient [38]. Filled vesicles attach to the terminal plasma membrane in a process known as docking. ATP6V1A and ATP6V1B2 identified in our studies as heavily phosphorylated have been isolated from rat docked synaptic vesicle fractions indicating that these subunits play a key role not only in neurotransmitter loading into synaptic vesicles, but also in the docking mechanism [37]. Interestingly, although the same protein was affected by all drugs, lithium, or valproate treatment resulted in phosphorylation of ATP6V1A, while paliperidone resulted in phosphorylation of ATP6V1B2.

Docked vesicles undergo a step called priming, which can be defined as acquiring the capacity to respond to calcium influx. Priming produces a ‘readily-releasable’ pool of vesicles [39]. When an action potential invades the synaptic terminal, calcium entry through voltage-gated channels can trigger fusion of readily-releasable vesicles with the pre-synaptic plasma membrane and the release of their contents in the synaptic cleft. We have previously demonstrated that SNAP25, a SNARE protein key for exocytosis, is upregulated by valproic acid treatment [3].

The functional significance of pre-synaptic plasma membrane V-ATPase subunits is still a matter of debate [40]. However, V-ATPase was recently shown to be involved in stimulation-dependent alkalinization of the cytoplasm, where it may be involved in regulating endocytosis [41]. It has also been suggested that the hydrophobic V0 membrane sector of the V-ATPase is directly involved in membrane fusion and neurotransmitter release [38].

As lithium, valproate and paliperidone influence dopamine and serotonin concentrations, it is possible to hypothesize that the ratios of serotonin to dopamine at the synapse might influence mitochondrial transport [42], [43], [44]. Serotonin and dopamine have opposite effects on mitochondrial movement in terms of direction [45]. Serotonin promotes anterograde movement towards axons and dendritic terminals while dopamine inhibits mitochondrial transport [14]. Mitochondrial trafficking has just now been linked to changes in the activity of neurons modulated by serotonin and dopamine [46], [47], [48]. Therefore, our hypothesis regarding the effect of mood stabilizers (and antipsychotics) extends beyond the traditional mode of action of these drugs on receptor binding and synaptic plasticity. We propose that each mood stabilizer or combination of drugs will generate a characteristic ratio of serotonin/dopamine at the synapse that will be reflected by the binding affinity ratio of 5HT2A/D2 receptors. Therefore, each drug or drug combination will have different influences on actin and tubulin polymerization and consequently on the direction and extent of mitochondrial movement (Figure 4C).

At the post-synapse, the cytoskeleton has been characterized in detail in dendritic spines where F-actin forms the majority of filaments implicating actin phosphorylation as a key mechanism for plasticity [49]. Filaments extend into the dendritic spine head from the synaptic junction (Figure 4D). An additional network of filaments is present throughout the spine head. We previously identified neurofilament triplet H (NFH) as up-regulated in response to chronic valproate treatment [3]. In our current study, we identified a similar neurofilament protein (AINX) as heavily phosphorylated (>7 fold) after mood stabilizer treatment. A link between AINX and BD has been recently made [50]. Both AINX and NFH occur typically in post-synaptic fractions. We hypothesize that these proteins will contribute to the formation of a network of filaments dedicated to maintain plasticity and promote mitochondrial migration and docking at post-synaptic heads (Figure 4D).

As described by Li mitochondrial numbers increase at the synapse with increased synaptic activity [51]. Interaction between tubulin and actin networks results in more effective transport of mitochondria [52] (Figure 4E). Orientation of tubulin into parallel microtubules and the assembly of actin into helical structures are determined by changes in polarity, which in turn are modulated by the presence of negatively charged phosphate groups. Association of mitochondria with neurofilaments, actin, and tubulin suggests that these cytoskeletal proteins contribute not only to mitochondrial movement but also to docking [53]–[57].

We observed an increase in mitochondrial staining with more defined processes after mood stabilizer treatment in rat primary neuronal cultures, when compared to staining in the controls. Careful monitoring of the staining reaction revealed detrimental effects to the cells when high concentrations of the dye or long incubation times were used. Other investigators have reported detrimental effects of MitoTracker Red® in cell culture [58]. Mitochondrial uptake of MitoTracker® dyes can also be influenced by loss of membrane potential and increased oxidant burden. Furthermore, these dyes inhibit respiration at high concentrations [59]. Therefore, monitoring cell viability with the use of Calcein was necessary to optimize the staining process in terms of concentration and duration of exposure to avoid detrimental effects on the cells. Light exposure was carefully minimized to avoid artifacts. Although the limitations of using MitoTracker® dyes are obvious, they proved to be a valuable method to determine mitochondria-associated changes and distribution in cell culture after drug treatment.

MitoTracker Red ® is internalized in response to changes in mitochondrial membrane potential. The increased staining observed after mood stabilizer treatment could be explained as the result of either increased mitochondrial numbers or increased MitoTracker Red ® uptake. In both instances, these changes are related to mitochondrial movement. Movement has been associated with a disruption of mitochondrial membrane potential [60]. Marked elevations in mitochondria-associated probe fluorescence due to changes in mitochondrial membrane potential have also been observed in cells engaged in active movement [61]. In a separate study, 90% of mitochondria in neurons stained with the mito-potential sensing dye JC-1 were transported towards the growth cone, while 80% of those with low potential were transported back towards the cell body [62]. Our studies using rat PFC tissue slices support our observations in cell culture. In particular, animals treated with lithium or paliperidone showed increased mitochondrial staining with MitoTracker Red®, suggesting changes in mitochondrial membrane potential in this tissue or the presence of larger mitochondrial numbers.

Our studies revealed longer filamentous mitochondria present in the lithium- or paliperidone-treated cells characteristic of rapid movement and/or mitochondrial fission. Mitochondrial fission facilitates re-distribution of mitochondria in response to local changes in the demand for ATP and allows for disposal of faulty mitochondrial fragments through mitophagy [63]. It is logical to hypothesize that mitochondrial fission will also be involved in the mechanism of action of drugs that increase synaptic activity, if these drugs promote mitochondrial migration and improve mitochondrial function.

Throughout their traffic through the axon, mitochondria can quickly switch between anterograde and retrograde movement, and their net direction has been shown in isolated neurons to result primarily from modulation of the fraction of time spent moving anterogradely [64]. In addition, they can be shifted between moving and stationary states by changing axonal growth or intracellular signaling [64]–[66]. Several perturbations can halt mitochondrial movement throughout the axon, including treatment with drugs that uncouple the proton gradient across the inner membrane and that inhibit complex V (without inhibiting electron transport at complex III) [62], [67]. Cell culture studies showed that valproate had a profound detrimental effect on mitochondria (results not shown). Other groups of investigators have made similar observations [68]. Our results indicate that mood stabilizers promote migration of large numbers of mitochondria to the synapse.

In terms of mitochondrial function, common proteins found phosphorylated (although effects were observed in different subunits depending on the drug) included UQCRC1, ATP6V1A, and ATP6V1B2. Our previous results indicated that proteins from complex I and V of the electron transport chain were differentially expressed in synaptoneurosomal preparations of rat PFC. However, we did not find differential expression of proteins from complex III under the parameters chosen in our previous studies [3]. Phosphorylation of UQCRC1 increased after treatment with all drugs tested. Complex III or bc-1 complex is involved in catalyzing the reduction of cytochrome c by oxidation of coenzyme Q (CoQ) and the concomitant pumping of 4 protons from the mitochondrial matrix to the intermembrane space. UQCRC1 also mediates formation of the complex between cytochrome c and c1. It is possible that phosphorylation of UQCRC1 results in uncoupling of the proton gradient across the mitochondrial inner membrane regulating mitochondrial movement. Decreases in expression of UQCRC1 have been reported in *post-mortem* PFC brain tissue of depressed patients [69]. The implications of these findings on BD and other mood disorders need to be further explored.

Paliperidone had an effect on the phosphorylation state of folding proteins HSP7C, HSP90B, and GRP78, as well as some regulatory proteins such as HOME1 (also called HOMER1) and PKC inhibitor protein 1(1433Z) (also called KCIP-1 or phospholipase A2). HSP90B and GRP78 have been related to ATP production. Increased levels of these proteins have been linked to diabetic muscle along with ATP synthase beta subunit (ATP5B) [70]. Protein 1433Z was highly phosphorylated after paliperidone treatment. Its target, PKC, plays a major role in regulation of both pre- and post-synaptic neurotransmission. Excessive activation of PKC results in symptoms related to bipolar disorder [71]. Another protein, SNAP25, has been recently identified as the target of PKC phosphorylation critical to PKC-dependent incorporation of synaptic *N*-methyl-d-aspartate (NMDA) receptors and relevant to synaptic plasticity [72]. We identified SNAP25 in our previous proteomics studies as one of the proteins whose expression was affected by treatment with valproate [3]. The interaction between SNAP25 and 1433Z remains to be studied. HOME1 is a post-synaptic density scaffolding protein that has mainly been associated with schizophrenia and cocaine addiction in African Americans [73], [74], [80].

The most unusual structural protein identified in our studies as heavily phosphorylated was a protein homologous to DESP. This protein is an obligate component of functional desmosomes and acts by anchoring intermediate filaments to desmosomal plaques. Desmosomes are intercellular junctions that tightly link adjacent cells. The N-terminus of DESP is required for localization to the desmosome and interacts with cadherins and the N-terminal region of plakophilin 1 and plakoglobin [75]. The molecular architecture involves an alpha-helical domain able to mediate homodimeric coil-coil formation. It is flanked by a non-alpha helical amino terminal domain that may harbor actin binding domains (ABD) and/or microtubule binding domains (MBD) and a non-alpha helical carboxy terminal domain that in addition to intermediate filament (IF) binding sites may contain variable numbers of DESP-type repeat domains [75]. Desmoplakin–intermediate-filament (DIF) interactions are modulated by the phosphorylation of a specific serine residue that is present in the carboxy-terminal domain. Phosphorylation can alter trafficking of cytoplasmic DESP into newly formed cell contacts [76]. Although members of the cadherin family have been linked to synapse morphogenesis and plasticity [77]–[79] the role of DESP or homologous proteins at the synaptic level has not been described. To our knowledge, we are the first group to report DESP homologous protein phosphorylation at the synaptic level in response to mood stabilizers. While the traditional function of DESP has been associated to the stabilization of desmosomes, we postulate that the function of this protein homolog goes beyond this traditional role and that DESP homologs may play an essential role to maintain synaptic plasticity and regulate homeostasis. Given its close association with cadherins and their critical roles in dendrite spine morphogenesis, synapse formation and remodeling [79], [81]–[85], we also postulate that DESP homologs should be considered as possible targets for cognitive and affective disorders.

To summarize our findings and proposed model for the effects of mood stabilizers on mitochondrial transport: It is clear that mitochondrial dysfunction and transport are involved in the mechanism of action of mood stabilizers and antipsychotics [87], [88], [89], [90], [91]. Mitochondrial recruitment and anchoring at pre- and post-synaptic terminals as a result of lithium, valproate, and paliperidone treatment seems to occur through a mechanism that involves phosphorylation of ACTB, ACTS. TBA1B, MYH1, MYH2, MYH6, AINX, and NFH, all connected through DESP-homologous proteins. Direction of this movement is regulated by differential ratios of dopamine and serotonin characteristic of each particular mood stabilizer or drug combination. Long range mitochondrial transport is facilitated by microtubules, kinesin, and dynein motors. These motors use ATP hydrolysis to generate movement [14]. Long range movement directs mitochondria along axons towards the synaptic terminals. Short range and local movement are facilitated by myosin along actin fibers [86]. Short range local movement might be used to fine-tune the position of mitochondria near high energy demand domains where they become anchored.

At the pre-synapse, mitochondrial anchoring close to synaptic vesicles would provide ATP; facilitate calcium homeostasis; and neurotransmitter loading, docking, and release. At the post-synapse, mitochondrial anchoring close to GSK3 would result in mitochondrial control of calcium ion concentrations and improved synaptic plasticity. GSK3 in turn would also be implicated in the regulation of mitochondrial movement, which is perhaps one of the reasons mood stabilizers seem to affect GSK3 directly or indirectly (Figure 4B). Improved mitochondrial function at synaptic terminals restores defects in neurotransmission. These changes might be transient due to the effects of mitochondrial fusion and fission as these organelles age and are recycled. Additional research is needed to explain the effects of mitochondrial fission and fusion in the mechanism of action of mood stabilizers.
